# 77 One Year Evaluation of a Targeted Candida auris Screening Program at a Comprehensive Cancer Center

**DOI:** 10.1017/ash.2026.10505

**Published:** 2026-06-23

**Authors:** Katie Thure, Dipen Patel, Christopher Evans, Simone Godwin

**Affiliations:** 1 Tennessee Department of Health; 2 Tennessee Department of Public Health; 3 TN Department of Health

## Abstract

**Background:** The National Healthcare Safety Network (NHSN) Standardized Antimicrobial Administration Ratio (SAAR) is a widely used metric for benchmarking antimicrobial use and supporting antimicrobial stewardship efforts. The Centers for Disease Control and Prevention (CDC) Priority Core Elements of Hospital Antibiotic Stewardship Programs offer a complementary, framework-based approach to evaluating stewardship infrastructure and practices. Understanding the relationship between implementation of Priority Core Elements and SAAR performance may help clarify how structural stewardship components translate into measurable antimicrobial use outcomes. Tennessee Department of Health explored the association between NHSN SAARs and Priority Core Elements to better inform stewardship program evaluation and improvement strategies. **Methods:** Using 2023 NHSN data, TDH evaluated the relationship between all-antibacterial SAARs by overall Priority Core Element and by interventions that comprise each element. Aggregate SAAR data from qualifying adult units was utilized to create a hospital wide SAAR. Specific Priority Core Element components—including leadership, accountability, pharmacy expertise, action and reporting were analyzed. The tracking priority was excluded as participating hospitals had to submit antimicrobial use data to generate a SAAR. Associations between SAAR performance and presence of interventions for each included Priority Core Element were analyzed using t-tests in SAS 9.4. **Results:** Mean all-antibacterial SAAR declined with greater attainment of priority Core Elements (1.13, 1.07, and 1.01 for 0–2, 3–4, and 5 elements, respectively), though the difference was not statistically significant (p=0.31). Hospital leadership commitment was not associated with SAAR differences. Hospitals meeting the Accountability Core Element demonstrated significantly lower SAARs than those that did not (1.02 vs 1.14; p=0.035). Hospitals with physicians who completed infectious diseases fellowships (0.96 vs 1.10; p=0.004) or pharmacists who completed stewardship certificate programs (1.00 vs 1.11; p=0.027) demonstrated lower SAARs. Action and Reporting Core Elements showed trends toward lower SAARs. Facilities implementing treatment recommendations with both prospective audit and feedback and preauthorization had significantly lower SAARs (1.00 vs 1.13; p=0.014). **Conclusion:** Implementation of accountability and targeted stewardship training was associated with lower all-antibacterial SAARs, suggesting that specific structural and personnel-focused interventions may improve antibiotic use. Action and Reporting Core Elements demonstrated trends toward lower SAARs, but these were not statistically significant. Findings underscore the potential value of Priority Core Elements in guiding stewardship strategies; however, limited sample size may have reduced the ability to detect additional associations. Larger, multistate studies are needed to confirm these relationships and further elucidate how comprehensive Core Element implementation impacts antimicrobial use outcomes.

